# Therapy Sessions as Part of the Prenatal Care Programme

**DOI:** 10.1192/j.eurpsy.2025.688

**Published:** 2025-08-26

**Authors:** A. Mihai, M. R. Isabela

**Affiliations:** 1 Psychiatry, University of Medicine, Pharmacy, Science and Technology George Emil Palade Targu Mures; 2 Psychiatry, Mureș County Hospital, Targu- Mures, Romania

## Abstract

**Introduction:**

Prevention is key to maintain a healthy lifestyle and to ensure a positive outcome of any given condition, especially in such a vulnerable one, as is pregnancy. The progress made in prenatal care in the last century is astounding and professionals nowadays are able to provide state of the art investigations and inventions throughout pregnancy and even before, but there is a dire need to address the mental health approach of the soon-to-be mothers. Since studies have shown the link between mother’s emotions and the child’s well-being, providing support in order to maintain a good mental health status should be part of the prenatal care programmes.

**Objectives:**

The initial hypothesis is that pregnancy is a delicate period for the mother to be, therefore anxiety levels can be high or prenatal depressive episodes can occur in the absence of good psychological support. We aim to prove that integrating psychotherapy as part of the prenatal care programmes has benefits for both mothers and child and can significantly reduce the struggle of battling these symptoms on one’s own.

**Methods:**

We launched an online questionnaire on groups and sites that targeted pregnant women or women who have just given birth. The questions were designed to address the anxiety and/or depressive symptoms throughout the pregnancy, the support they received from the health care providers and the impact these had on how they handled the pregnancy period. Anxiety symptoms were assessed using the HAM-A scale, meanwhile for depressive symptoms we used PHQ9 questionnaire. The study was conducted anonymously and was approved by the local ethics committee.

**Results:**

**We received 200 answered questionnaire, from which 189 were valid and relevant to the study.**

The results showed that the majority of pregnant women (83%) have struggled with anxiety and/or panic attack throughout the pregnancy and the prenatal care appointments proved insufficient to alleviate their concerns. Most of them (94%) turned to the internet for answers, which was an aggravating factor for the symptoms. The impact these symptoms had on their pregnancy varied from tensions between them and their partners, insomnia, feelings of worthlessness and irritability to moderate depressive episodes. Almost all the respondents (91%) have answers affirmatively to the proposal to undergo a few therapy sessions to manage these symptoms, if they were to be part of the prenatal care programme.

**Conclusions:**

Pregnant women are at risk for various mental health issues which can be prevented with proper care, thus addressing further risk for both the mother and the baby. The need for integrating therapy in prenatal care programmes could have a great impact on the outcome of the pregnancy and even in women desire to bear children.

**Disclosure of Interest:**

None Declared

